# Stable Surface Modification by Cold Atmospheric-Pressure Plasma: Comparative Study on Cellulose-Based and Synthetic Polymers

**DOI:** 10.3390/polym15204172

**Published:** 2023-10-20

**Authors:** Alina Silvia Chiper, Gabriela Borcia

**Affiliations:** Iasi Plasma Advanced Research Center (IPARC), Faculty of Physics, Alexandru Ioan Cuza University of Iasi, Blvd. Carol I No. 11, 700506 Iasi, Romania; alina.chiper@uaic.ro

**Keywords:** cellulose, polymer, plasma treatment, surface modification

## Abstract

This study’s aim is a comparison of the plasma-induced effects on polymers exposed in helium and argon gaseous environments in a pulsed dielectric barrier discharge at atmospheric pressure. Cellulose-based and synthetic polymers are tested with regard to a range of parameters, such as wettability, adhesion, surface energy and polarity, the oxygen amount in their structure, and surface morphology. The surface properties are analyzed by contact angle measurements, X-ray photoelectron spectroscopy, and scanning electron microscopy images. The results point to the efficient and remarkably stable modifications of the plasma-exposed surfaces, such as their enhanced adhesion, surface energy, and oxygen incorporation. Additionally, plasma provides significant oxygen uptake in cellulose-based materials that bear already prior to treatment a high amount of oxygen in their structure. The comparison between the properties of the non-permeable, homogeneous, smooth-surface synthetic polymer and those of the loosely packed, porous, heterogeneous cellulose-based polymers points to the different rates of plasma-induced modification, whereby a progressive alteration of cellulosic surface properties over much larger ranges of exposure durations is noted. Present experimental conditions ensure mild treatments on such sensitive material, such as paper, and this is without alterations of the surface morphology and the physical degradation of the material over a large range of treatment duration.

## 1. Introduction

Polymers are complex materials with various properties that result from a combination of their chemical structure, chain structure, and the presence of functional groups with different polarity in the main chain and/or as pendant groups, as well as their degree of crystallinity [1]. They comprise a comprehensive range of bulk properties, rendering polymers the most versatile materials for applications. Nonetheless, the surface properties of polymers, which are paramount in practice as they govern the compatibility and interaction at the interface of the material and its working environment, cover a rather limited range of variation. This limited range is mainly associated with the intrinsic hydrophobic character of polymer surfaces. Therefore, the techniques allowing for the tuning of polymer surface wettability and polarity are of continuous interest.

In this general context, paper-based materials—having cellulose as a major component—represent a particular type of polymer which is used in a wide variety of applications that relate closely to surface wettability, such as inkability, printability, particle immobilization, etc. [2,3,4,5,6]. To this end, surface modification methods are used to appropriately tune surface properties, as well as to preserve the bulk properties, of such sensitive, porous material compared to compact film polymers, and plasma treatment is demonstrably a convenient tool for this purpose [7].

The advantages of the plasma techniques used for the surface modification of polymers arise from plasma’s various components, such as excited and ionized particles, photons, and radicals. These constituents possess the energy necessary to induce chemical reactions, both in the plasma volume and at its interface with solid surfaces [8,9,10,11,12]. Importantly, plasma treatment is recognized as modifying only a few surface and subsurface layers while preserving the unaffected bulk material [13,14].

The plasma-induced modifications of polymers relate to several processes which are common for most materials. These processes start with surface cleaning by ablation of the small molecules and of the various contaminants with low molecular weight. This is followed by a break of the weak chemical bonds and radical formations, which then trigger secondary reactions, such as functionalization and intermolecular crosslinking [13]. Nonetheless, these processes are usually achieved in a complex combination, and the final surface properties of the plasma-treated material may be unique for each type of polymer. The final properties depend on all of the factors involved, such as polymer structure parameters, plasma exposure conditions, and—last but not least—post-treatment recovery.

In this frame, various types of plasma were used in order to obtain a controlled and stable modification of paper-based materials. The general results pointed to an enhanced surface wettability and a measurable level of oxidation after exposure to reactive gases [3,4,5,15,16,17,18,19,20], which are also associated with increased adhesion for the subsequent surface deposition of functional layers [21] and composite manufacturing [22].

Nonetheless, in most cases, the treated paper-based surfaces showed, to a certain extent, a tendency toward being restored, at least in terms of loss of the hydrophilic character, thus showing what is called aging [18,23,24,25].

Additionally, although plasma presumably modifies only the topmost layers—thus preserving the material bulk—in some cases, plasma exposure triggers an obvious alteration of the surface morphology and sometimes even etching, which affects the micro- and nanostructure at its depth [18]. This results in a wettability modification that is related to a physical effect of plasma, one that is enhanced by the porous nature of paper-based materials [18].

Taking this into account, in this paper, a comparison of the plasma-induced effects on two types of polymers with distinct sources is carried out. These polymers are exposed to helium and argon gaseous environments in a pulsed dielectric barrier discharge (DBD) at atmospheric pressure. The tested materials are paper (P) (which has cellulose, a natural polymer, as its main component), fluorinated paper (FP) (which is derived from cellulose that is modified by the addition of fluorine), and polysulfone (PSU) (which is selected as a representative synthetic polymer due to it having a compact non-permeable structure when compared to the more loosely packed and porous cellulose-based paper). All of the polymers tested in this study contain cyclic structures. The cellulose-based polymers contain an anhydroglucose ring [26,27,28], while polysulfone contains an aryl functional group that is derived from an aromatic hydrocarbon ring (C_6_H_6_) [29]. It is noteworthy that the aryl group has an intrinsically higher chemical resistance to modification, thus allowing one to assess the plasma capability in altering such materials.

The properties of polysulfone, such as high thermal stability, low flammability, mechanical strength, transparency, and chemical resistance, make it desirable for many applications [30]. The filtration industry uses polysulfone membranes in different forms: flat sheets or hollow fibers for water purification, gas separation, hemodialysis, or seawater desalinization [30,31]. However, the use of polysulfone membranes is restricted because of their hydrophobic character. Therefore, many studies have been conducted to enhance their hydrophilic character [32,33,34].

Due to its abundance, cellulose and its derivatives are also widely used in different applications such as dialysis membranes, artificial kidney membranes, wound dressing, and bandages [35]. The ability of cellulose to absorb is essential in medical applications [36]. In addition, many studies have been conducted to delay the aging process of valuable archival documents for the better preservation of cultural heritage [37]. Furthermore, cellulose-based polymers offer an excellent substitute for synthetic polymers, and the processing of cellulose-based composites currently has the attention of researchers.

The objective of this paper is to explore and compare the surface properties of cellulose-based and synthetic polymers exposed to atmospheric-pressure DBD plasma generated using either He or Ar as working gas.

To comprehensively analyze the effects of plasma on the polymer surface, it is necessary to thoroughly characterize the plasma source, which serves as the “tool” for modifying polymer surface properties. It is important to note that the outcomes of plasma exposure are closely related to the properties of the active species created in the plasma volume and at the plasma–material interface [38]. Given that the plasma source has been extensively characterized through measurements of electrical and spectral discharge parameters [38,39,40,41], this investigation is centered on the analysis of the polymer surface properties. 

The analyzed surface properties include adhesion work, surface free energy, and its components determined through contact angle measurements, surface chemical composition assessed via X-ray photoelectron spectroscopy (XPS), and surface morphology captured in scanning electron microscopy (SEM) images.

## 2. Experimental Setup and Methods

A dielectric barrier discharge (DBD) system with a symmetrical electrode configuration, as described in detail previously [38,40], is employed to produce a cold (non-equilibrium) atmospheric-pressure plasma in an enclosed chamber. The inter-electrode gap measures 3.0 mm. Positive voltage pulses, with a repetition frequency of 2 kHz, pulse width of 30 μs, and amplitude of 4.0 kV, are applied to initiate the discharge. These experiments are conducted at atmospheric pressure using high-purity inert gases (He and Ar, 99.996%). The working gas is continuously supplied into the inter-electrode gap at a flow rate of 3000 sccm gas, without preliminary vacuum pumping. The plasma exposure time ranges from 5 s to 120 s.

To assess the impact of the discharge gases (He/Ar) on the surface properties of plasma-treated polymers, all structural and operational parameters of the plasma source are kept constant throughout the experiments. Notably, the energy transferred to the discharge system per voltage pulse is 0.6 mJ [38]. Consequently, the discharge electrical power remains unchanged at 1.2 W, regardless of the working gas used (He/Ar). 

The plasma treatments are conducted on two categories of test materials: paper (P) and fluorinated paper (FP), both of which fall into the category of cellulose-based polymers. Additionally, polysulfone (PSU) (Goodfellow Ltd., Huntingdon, UK) is used as a synthetic polymer. P and FP foils (Ceprohart S.A. Brăila, Romania) are primarily composed of bleached sulfate cellulose derived from equal proportions of hardwood and softwood. In the case of FP foils, there is the inclusion of calcium carbonate, a super-hydrophobic fluoropolymer, and various additives (a cationic polymer as a retention agent and alkyl ketene dimer as a sizing agent). Notably, P foils are specially formulated to possess the same composition as FP, with the exception of the super-hydrophobic fluoropolymer.

The surface analysis is performed through contact angle measurements, X-ray photoelectron spectroscopy (XPS), and scanning electron microscopy (SEM).

Contact angles are determined using the sessile drop technique with a drop volume of 1 μL. We use an automated system for capturing the drop images which is equipped with a digital camera (Optika 4083.B5, from Optika S.r.l., Ponteranica, Italy) and a PC-based control, acquisition, and data processing system. Due to the significant reduction in contact angle when liquid drops are dispensed on the plasma-treated cellulose-based foils, we have adapted the data acquisition method to accommodate this particular situation. Following the deposition of a liquid drop on the surface, images are captured every second until the contact angle stabilizes, with a maximum observation duration of 50 s. Static contact angles are determined using the open-source software ImageJ (Microsoft Java 1.1.4.), and the presented values represent the average of at least five measured values obtained as described earlier.

The relative increase in the adhesion work, defined as

∆W_a_/W_a_ = (W_a(treated)_ − W_a(untreated)_)/W_a(untreated)_, (1)

is used as a control parameter for the plasma-induced effect on the polymer surface, where the adhesion work is [42]:

W_a_ = γ_L_(1 + cos θ), (2)

where θ is the contact angle, and γ_L_ is the surface tension of the test liquid (water or glycerol) presented in Table 1 [43].

Furthermore, the values in Table 1 are used to calculate the surface energy (γ_S_) and its polar (γ_S_^p^) and dispersive (γ_S_^d^) components using the OWRK method (Owens, Wendt, Rabel and Kaelble model) [43,44].

Then, the total surface energy is

γ_S_ = γ_S_^d^ + γ_S_^p^ (3)

and the surface polarity is defined as:

γ_S_^p^/γ_S_ = γ_S_^p^/(γ_S_^d^ + γ_S_^p^). (4)

The XPS spectra are recorded on a PHI-Ulvac VersProbe 5000 spectrometer (Φ ULVAC-PHI, Inc., Chigasaki, Kanagawa, Japan), using the Mg Kα line (hν = 1253.6 eV), at 45° take-off angle. The energy scale is calibrated using the value of 285.0 eV of the hydrocarbon C1s core level. The peak envelopes are curve-fitted using mixed Gauss–Lorentz component profiles.

The evaluation of the sample morphology is performed using a Quanta 250 (FEI) Scanning Electron Microscope (Thermo Fisher Scientific, Waltham, MA, USA), operating in high vacuum mode, at a high voltage of 20 kV and an emission current of around 100 µA. The samples are attached to 0.5″ aluminum specimen stubs (G301—Agar Scientific, Stansted, UK) using 12 mm conductive adhesive carbon discs (G3347—Agar Scientific). The electron micrograph acquisition is performed using the backscattered electrons detector (BSED) in z-contrast analysis mode. The analysis conditions for image recording include a resolution of 4096 × 3536 pixels, a dwell time of 2 ÷ 5 µs, and a spot size of 4. No post-processing of the recorded images is performed. Beforehand, a thin layer of copper is deposited by magnetron sputtering on the non-conducting samples to avoid the electrical charging under electron bombardment.

## 3. Experimental Results and Discussions

The surface properties assessed for the three test materials include adhesion work, surface free energy, and its components determined through contact angle measurements, surface chemical composition via X-ray photoelectron spectroscopy (XPS), and surface morphology examined with scanning electron microscopy (SEM) images.

### 3.1. Contact Angle Measurements

The contact angle undergoes changes for all plasma-treated surfaces investigated, indicating that the plasma imparts a hydrophilic character compared to the untreated hydrophobic surfaces. Notably, cellulose-based materials P and FP exhibit a distinct behavior, with the contact angle decreasing during measurement due to their absorbent properties. Similar results were reported in [2,3]. This decrease occurs exclusively in the treated materials, not in the untreated ones, suggesting that plasma exposure triggers the enhanced absorbency.

**Figure 1:**
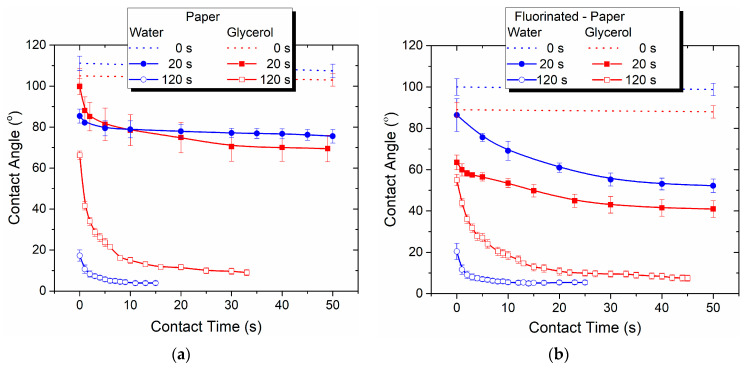
Typical temporal changes in contact angles for water and glycerol droplets dispensed on surfaces of (**a**) paper and (**b**) fluorinated paper, untreated and treated in He-DBD plasma for varying durations (20 s and 120 s). The moment of liquid drop deposition on the surface is indicated as 0 s.

In this regard, we analyze the temporal evolution of droplets dispensed on cellulose-based foils by measuring the contact angle as a function of the contact time between the droplet and the surface. Figure 1a,b illustrate the typical temporal changes in the measured contact angle for liquid droplets (water and glycerol) dispensed on P and FP foils treated in He-DBD plasma. These figures also demonstrate that the contact angle remains constant over time for the untreated samples. However, post plasma treatment, the contact angle primarily fluctuates during the first 10–30 s of contact, dependent on both the treatment duration and the test liquid used, after which it stabilizes. The longer the treatment, the quicker water/glycerol molecules diffuse into the cellulose structure. Similar results were reported in [2]. 

Interestingly, the contact angle corresponding to the equilibrium at the interface between the material and the liquid depends on the duration of plasma exposure. Measurable values are observed, and complete absorption only occurs with prolonged exposure. For instance, even a relatively short He plasma treatment of 20 s results in contact angles with water of 75.6° ± 3.3° on P (compared to 107.4° ± 3.3° for the untreated) and 52.3° ± 3.2° on FP (compared to 98.8° ± 3.1° for the untreated). It is only when the exposure duration exceeds 120 s that we can anticipate complete diffusion of water droplets into the cellulose structure, resulting in a contact angle approaching nearly 0° (Table 2). The absorption of the glycerol droplets is slower due to its higher viscosity. Our experiments show that, within the current plasma setup, 120 s represents the maximum duration of plasma exposure at which a measurable water contact angle is achieved.

An overall assessment reveals that the water contact angle, after 120 s of exposure to He-DBD, decreases significantly. For instance, the angle decreases from 107.4° ± 3.3° for the untreated P sample to its lowest value of 3.9° ± 1.0° (Figure 1a) and from 98.8° ± 3.1° for the untreated FP to its lowest value of 5.4° ± 1.0° (Figure 1b). In contrast, for the PSU samples exposed to He plasma for 20 s, the water contact angle varies from 83° ± 1.4° for the untreated sample to its lowest value of 36.5° ± 2.4° (Table 2). It is important to note that the PSU polymer does not exhibit absorbent properties.

Equilibrium contact angles are determined from the graphical representation of the temporal evolution (as in Figure 1). These angles are then used to calculate the adhesion work and the surface free energy of the cellulose-based polymers. 

**Figure 2:**
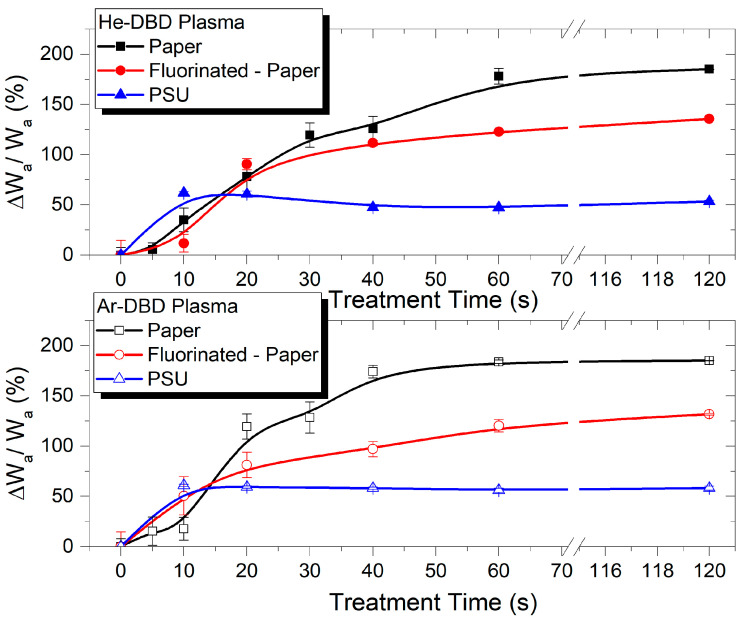
Relative variation in water adhesion work vs. treatment time for paper, fluorinated paper, and PSU samples treated in He-DBD plasma (**top**) and Ar-DBD plasma (**bottom**).

**Figure 3:**
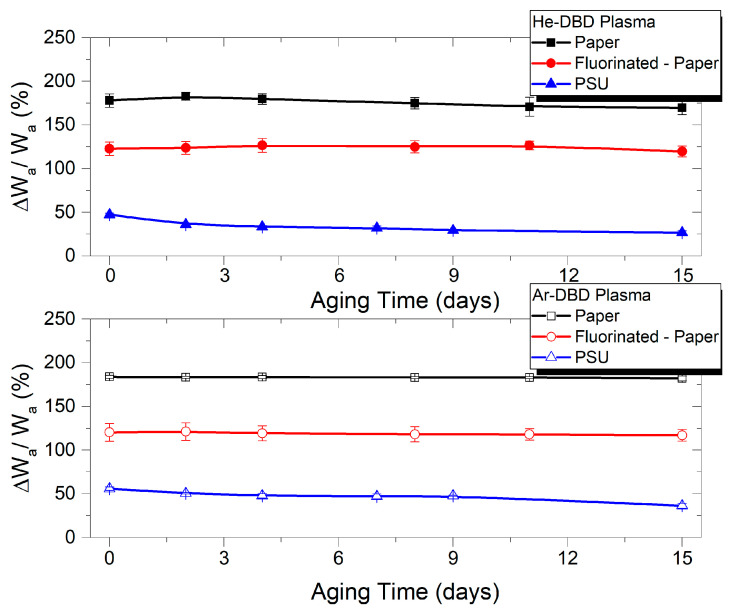
Relative variation in water adhesion work vs. aging time for paper, fluorinated paper, and PSU samples exposed to He-DBD plasma (**top**) and Ar-DBD plasma (**bottom**) for 60 s.

The reduction in contact angle corresponds to an increase in the water adhesion work W_a_ [42]. Consequently, we present the relative increase in W_a_ for the three test materials treated in the two inert gases in Figure 2 and Figure 3 as a function of the treatment time and the aging time, respectively.

The water adhesion work W_a_ for the untreated surfaces is 51 mJ/m^2^ for P, 62 mJ/m^2^ for FP, and 82 mJ/m^2^ for PSU. This order of values aligns with the hydrophobic character of these surfaces, with P < FP < PSU. 

The surface modification exhibits rapid changes within the initial 10–20 s of treatment for all three polymers, as shown in Figure 2. The relative increase in ∆W_a_/W_a_ is approximately 6% per second for PSU and around 2–4% per second for the two cellulose-based polymers. Following this, ∆W_a_/W_a_ stabilizes for PSU, reaching a limit value of about 55%, whereas it continues to increase to a substantial rate for P and FP. Saturation is only achieved for the cellulose-based polymers with extended treatment times (beyond approximately 60 s), with limit values of 135% for FP and 185% for P. Notably, the values for the same polymer surface treated in the two inert gases are very close (Figure 2).

The investigation of the post-processing recovery of the plasma-exposed polymers, as presented in Figure 3, demonstrates the remarkable stability of the modified surfaces. This applies to all three material types and exposure in both treating gases. The representation corresponding to the 60 s exposure duration, during which all three polymers reach their maximum level of modification in terms of wettability-related parameters, serves as an indicator of the efficient and stable surface modification. The surfaces exhibit an enhanced hydrophilic character and increased adhesion properties. Additionally, it underscores the ordering of these materials concerning the extent of adhesion modification as P > FP > PSU. This suggests that materials with lower initial adhesion are more prone to undergo a greater increase in their adhesion work.

**Figure 4:**
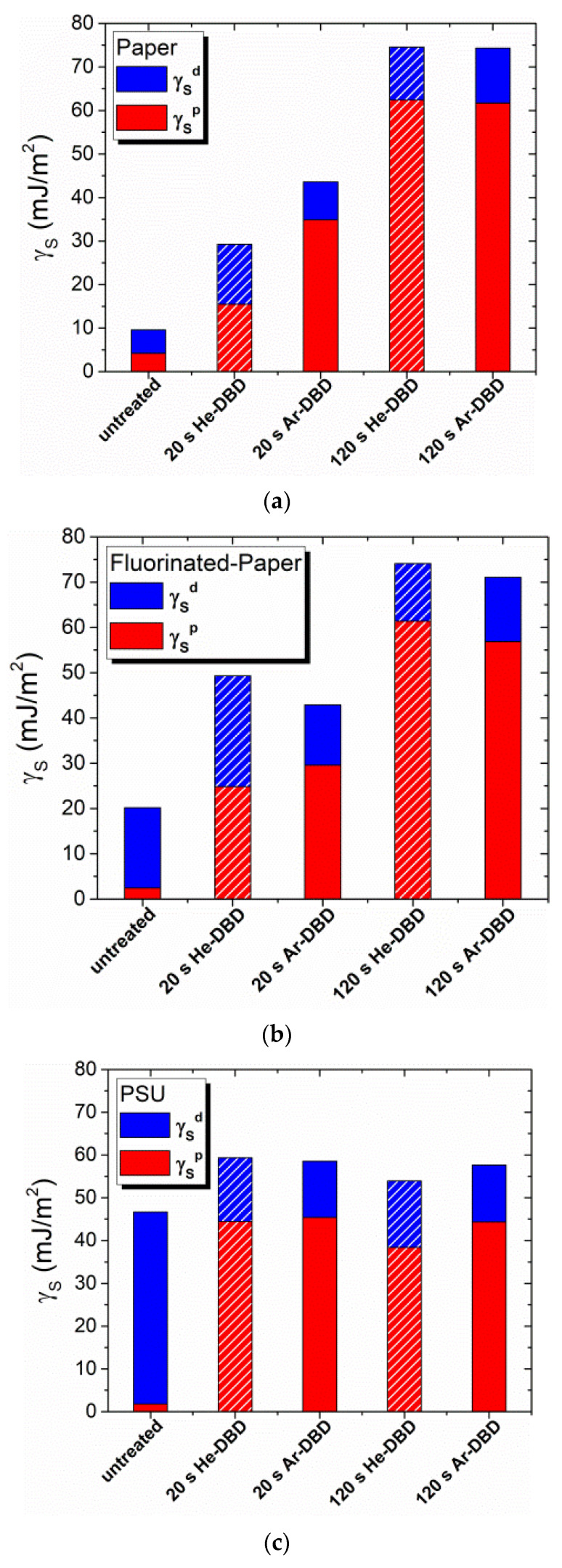
Polar (γ_S_^p^) and dispersive (γ_S_^d^) contributions to the surface free energy (γ_S_) of paper (**a**), fluorinated paper (**b**), and PSU (**c**) samples before and after plasma treatment in various experimental conditions.

In addition to the assessment of water adhesion work, we calculate the surface free energy (γ_S_), and its polar (γ_S_^p^) and dispersive (γ_S_^d^) components, for all three test materials [44], both before and after plasma exposure. The results of these evaluations are presented in Figure 4. In the initial state, PSU exhibits the highest surface energy at 46.6 mJ/m^2^, whereas P has the lowest surface energy at 9.6 mJ/m^2^. Once again, the plasma exposure leads to a reversal of the order. Thus, the treatment produces the most significant modification of the total surface energy (γ_S_) for the polymer with the lowest initial surface energy, which, in this case, is P (Figure 4a). Conversely, the lowest level of modification is observed in the polymer with the highest initial surface energy, which is PSU (Figure 4c). Notably, after 120 s of exposure, the surface energy (γ_S_) of cellulose-based materials converges to very similar values, about 71–74 mJ/m^2^ for FP and around 74 mJ/m^2^ for P, even though the initial values for untreated samples exhibit more variation. This suggests that the experimental arrangement ensures the maximum level of surface modification. On the other hand, the surface energy values for PSU emphasize that the limit of modification of this polymer is reached with shorter treatment time. For instance, the calculated γ_S_ values for PSU after 20 s and 120 s of exposure fall within the range of 54–59 mJ/m^2^, similarly for both working gases.

Table 3 provides a summary of the experimental results, specifically focusing on the surface polarity γ_S_^p^/γ_S_. The untreated surfaces exhibit varying levels of polarity, ranging from 0.038 for the synthetic polymer PSU to 0.437 for paper, with fluorinated paper falling in between at 0.121. Notably, the contribution of the polar groups to the total surface energy increases after treatment for all polymers.

Interestingly, despite pristine PSU having the lowest initial surface polarity, plasma-exposed PSU reaches the highest surface polarity (0.748 in He plasma and 0.775 in Ar plasma) at a relatively short treatment time (20 s). However, there is a tendency for this polarity to diminish with prolonged treatment (120 s). This observed behavior correlates with the XPS results, indicating that the optimal treatment time for the synthetic polymer is around 20 s. 

Similar to the trends observed for the other parameters discussed previously, cellulose-based foils reach their maximum surface polarity (0.80–0.84) at longer treatment times, specifically 120 s. 

The increase in surface polarity induced by plasma treatment is notable, indicating that the contribution of the polar component far surpasses that of the dispersive component. This behavior is attributed to the creation of polar groups with chemically bonded oxygen, as is further substantiated by the XPS results. The dominance of the polar component over the dispersive component in the case of plasma-exposed cellulose aligns with results reported in [45].

An important and interesting observation is that the maximum values of surface polarity γ_S_^p^/γ_S_ fall within a relatively narrow range for all three polymers, approximately 0.75–0.83 (Table 3). This finding indicates that the highest attainable surface polarity through plasma exposure is similar for materials with distinct chemical structures, polarities, and surface morphologies. 

### 3.2. Surface Chemical Characterization by XPS

**Figure 5:**
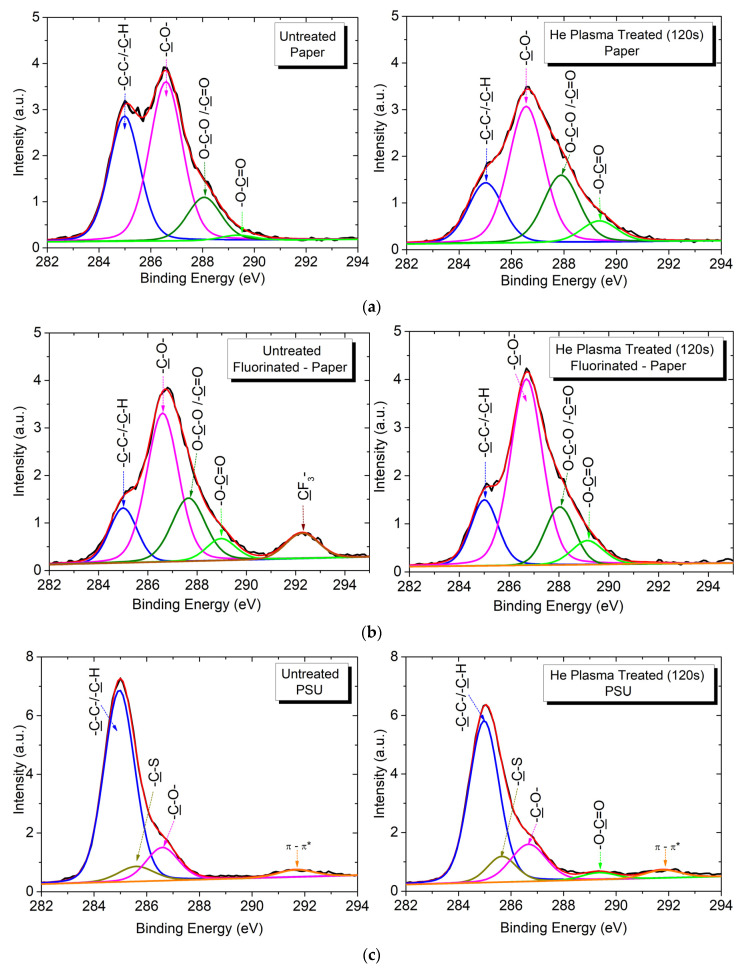
Typical deconvolutions of the high-resolution C1s XPS spectra for paper (**a**), fluorinated paper (**b**), and PSU (**c**) samples, untreated (**left**) and treated by He-DBD plasma for 120 s (**right**).

XPS is used to investigate the chemical structure of the polymer surfaces, with consideration of the fact that the most reactive species in the discharge is oxygen, primarily due to the inherent presence of residual air in the discharge chamber [38]. This occurs despite the discharge operating in a continuous flow of inert gas (He or Ar) injected into the discharge. The carbon 1s (C1s) XPS spectra of the test polymers (Figure 5) are fitted based on reference measurements [31,35,46,47] to identify and number the carbon chemical groups in increasing order of their binding energy (Table 4) [14,35,46,47,48,49].

Both cellulose-based polymers, P and FP, exhibit the same chemical groups, except that additional higher energy bonds are present in the fluorinated polymer FP. As a result, the C1s spectrum of untreated P is deconvoluted into four peaks, each assigned to one of four distinct categories of carbon bonds (Figure 5a). The first peak, labeled C1, corresponds to unoxidized carbon (–C–C–, –C–H) at 285.0 eV. The second peak, identified as C3 at 286.5 eV, is assigned to carbon singly bonded to oxygen (–C–O–). The third peak, C4, represents carbon with two oxygen bonds (O–C–O/–C=O) at 288.0 eV. Lastly, the fourth peak, C5 at 289.0 eV, is associated with carbon bearing three oxygen bonds (–O–C=O) [35,36,46]. Note that C3 and C4 correspond to the intrinsic components of cellulose, whereas C1 represents non-functionalized carbon [47]. Furthermore, in the case of FP, an additional peak, labeled C7 at 292.3 eV, is assigned to carbon bonded to fluorine (Figure 5b). Upon subjecting the cellulose-based samples to plasma exposure, the deconvolution of the C1s spectra reveals the presence of the same carbon groups, albeit with varying intensity when compared to the untreated samples. Notably, there is a visible reduction in the carbon–fluorine peak C7 (–CF_2_–/–CF_3_) in the FP polymer which tends to completely disappear with prolonged treatment (120 s).

The C1s spectrum of the pristine PSU is deconvoluted into three peaks (Figure 5c). These are assigned to carbon atoms bonded to hydrogen/carbon, sulfone, and ether groups at C1s core-level binding energies of 285.0 eV (C1), 285.6 eV (C2), and 286.5 eV (C3), respectively [31,48]. Additionally, a fourth peak, marked as C6, which corresponds to the low-energy π−π* shake-up transitions accompanying the core-level ionization, at around 291.8 eV [31,48], is present in both untreated and plasma-treated PSU samples (Figure 5c). In the case of plasma-treated PSU, there is a clear modification in the intensities of the peaks, along with the emergence of a new component. This new peak is located at 289.0 eV (C5) and corresponds to the carboxyl −O−C=O group created as a result of plasma exposure (Figure 5c).

Table 5, Table 6 and Table 7 provide data on the elemental surface composition, the oxygen content calculated from the survey scan XPS spectra (expressed as O1s/C1s), and the relative atomic composition of the carbon groups resulting from the deconvolution of the C1s high-resolution XPS spectra. The data demonstrate the progressive surface oxidation of the three tested polymers. This oxidation is attributed to the creation of new oxygen-bonded groups and/or the augmentation of the existing ones. Notably, this increase in the surface oxidation is reflected in the O/C ratio, which represents the proportion of oxidized to unoxidized carbon atoms on the surface. The values of O/C, presented in the same tables, are calculated as:

O/C = (C3 + C4 + C5)/C1 for paper,(5)

O/C = (C3 + C4 + C5)/(C1 + C7) for fluorinated paper,(6)

O/C = (C3 + C5)/(C1 + C2 + C6) for PSU.(7)

**Figure 6:**
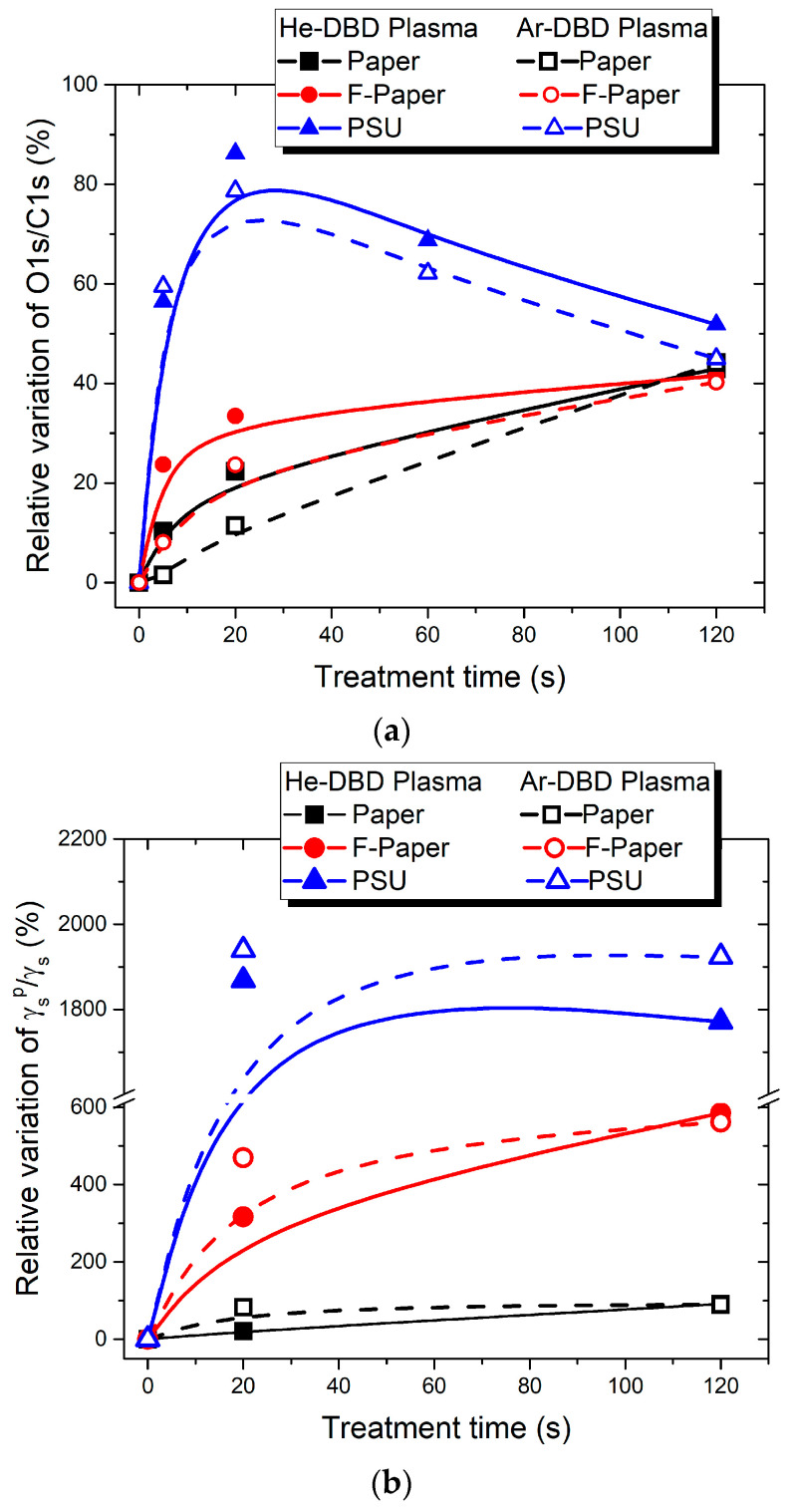
The relative variation of the O1s/C1s atomic ratio (**a**) and the surface polarity (**b**) vs. plasma treatment time for paper, fluorinated paper, and PSU. (The experimental data are plotted by beta-spline connected graph.)

An analysis of the data provided by the survey scans reveals that both cellulose-based materials initially possess a similar O1s/C1s atomic ratio, approximately in the range 0.50–0.51. However, it is worth noting that the total amount of oxygen in the pristine structures is different between the two, with FP containing a lower amount of oxygen (27 at. %) compared to P (33 at. %). Plasma treatment causes increased oxygen uptake as a function of the exposure time. The evolution with time is not identical and depends on the polymer type and treatment gas. However, for prolonged treatment (120 s), both materials reach a similar O1s/C1s ratio of approximately 0.72–0.73 (Table 5 and Table 6), and they also attain a similar oxygen content of around 40–41 at. % (Figure 6). This observation suggests that there is a limit to the amount of oxygen uptake in such cellulose-based materials. Indeed, it is important to note that the plasma treatment induces a significant oxygen uptake on materials such as cellulose-based polymers that prior to treatment already contain a significant amount of oxygen in their structure.

Nonetheless, the oxidation mechanism differs between the two polymers, as evident from the data obtained from the C1s high-resolution spectra. For the P samples, the quantity of unoxidized carbon (C–C/C–H) decreases as treatment time progresses as a part of these carbon atoms undergoes oxidation during plasma exposure. The XPS analysis reveals a reduced amount of unoxidized carbon C1 on the surface, along with an increased amount of carbon with two or three oxygen bonds, C4 and C5, respectively (Table 5). Similar results were reported in [36,50]. Consequently, the O/C ratio exhibits an increase of 60–70% compared to the untreated surface for shorter treatment durations (less than 20 s). However, with prolonged exposure (120 s), the level of oxidation surpasses a 100% increase (Table 5).

Conversely, the FP samples exhibit a different behavior. In this case, the quantity of unoxidized carbon (C–C/C–H), referred to as C1, remains relatively constant, and there is a decreased presence of carbon with two or three oxygen bonds, specifically C4 and C5 (Table 6). The increase in O/C during extended plasma treatment is attributed to a higher proportion of carbon with a single oxygen bond, C3, along with a reduction in the quantity of carbon bonded to fluorine, C7. It is worth noting that the degradation of fluorine-containing bonds due to plasma exposure has been reported in other papers, as in [51].

As a result, for FP, the O/C ratio experiences an increase of approximately 20–35% during shorter plasma treatment durations (less than 20 s). In contrast, with extended treatment times (120 s), the change in the level of oxidation is around 50–70%, and this can vary based on the type of the discharge gas used (Table 5). This indicates that the rate of O/C increase is comparatively lower for FP compared to P when subjected to the same exposure conditions.

The behavior of the PSU material differs from that of the two cellulose-based samples. While there is no evidence of a reversal in the process of oxygen uptake for both P and FP, even for prolonged exposure, in the case of PSU, the process appears to be reversible. It is interesting to note that, for PSU, the O1s/C1s and O/C ratios, which are calculated from two types of XPS spectra, are similar. This is because the synthetic polymer possesses a homogeneous structure, devoid of additives, and also has a flat, smooth surface which is ideal for XPS analysis. In contrast, P and FP have highly textured surfaces at the micrometric scale (as demonstrated later by SEM), and feature complex structures with additives and retention agents, making the analysis more challenging. 

In the case of the PSU surface, the O/C ratio experiences a significant increase, exceeding a factor of 2 for shorter plasma treatment times (5–20 s). However, for prolonged treatment, a clear limit of oxidation is reached. This extended exposure leads to a reversal in the surface functionalization, reducing the oxidation level (Table 7). This unique behavior in PSU is a result of the combined processes during surface modification, including surface oxidation and the ablation of low-weight volatile fragments. These processes occur at different rates and sometimes produce opposite effects. Over prolonged exposure to plasma, the ablation rate becomes more prominent, leading to a reduction in the level of oxidation. It is noteworthy that the PSU surface initially has the lowest oxygen content among the tested polymers, resulting in a lower maximum oxygen uptake from exposure to plasma compared to P and FP. Moreover, the level of oxidation in PSU is similar for both working gases.

The weaker oxygen incorporation and the observed reversal of the oxidation process after relatively short exposure times can be attributed to two key factors. Firstly, it is important to note the significant chemical resistance arising from the aromatic-based structure of PSU. Secondly, the unique surface morphology plays a vital role. PSU presents a compact and exceptionally smooth polymer film, while the cellulose-based samples exhibit a rough surface at the micrometric scale, characterized by a porous and more permeable structure. This distinctive surface structure increases the effective surface area of cellulose-based polymers exposed to the plasma discharge. As a result, the plasma can penetrate deeper into the material, inducing greater oxidation over longer treatment durations. 

At this point, we can establish a valuable correlation between the data acquired through XPS and contact angle measurements, as presented in Figure 6. This figure illustrates the relative changes in the O1s/C1s atomic ratio (a) and the surface polarity γ_S_^p^/γ_S_ (b) in comparison to their respective untreated states. 

We notice that the PSU surface attains its maximum level of oxidation at approximately 20 s of exposure, a finding supported by the XPS data (Figure 6a). This aligns closely with the peak in surface polarity, which we also observe at 20 s of plasma treatment (Figure 6b). Moreover, the water adhesion work also peaks at 20 s treatment time (Figure 2). These findings are in good agreement with those in [52]. However, as exposure times lengthen, the adhesion properties level out, and the surface polarity exhibits a slight tendency to reverse. Simultaneously, the level of oxidation undergoes a noticeable reversal. It is important to note that the reversal of polarity might be less apparent in contact angle measurements, which essentially probe only the first surface monolayer, whereas XPS examines a depth of about 50 Å.

Unlike PSU, the cellulose-based surfaces follow a different trend. Their properties continue to change throughout the entire range of exposure durations tested here, albeit at varying rates (Table 5 and Table 6 and Figure 6). The relative changes in the O1s/C1s atomic ratio (Figure 6a) correspond to those in γ_S_^p^/γ_S_ (Figure 6b), indicating a continuous increase in both parameters within the exposure duration range of 5 to 120 s.

In general, there are only minor differences between the results obtained for the two working gases. This behavior can be attributed to the fact that the discharge electrical power is kept constant throughout. In these conditions, when inert gases such as He or Ar are used, the primary reactive species in the discharge is typically oxygen due to residual atmospheric air. As a result, the differences in the plasma effects between He and Ar are relatively small in this context.

### 3.3. Surface Morphology Analysis by SEM Images

**Figure 7:**
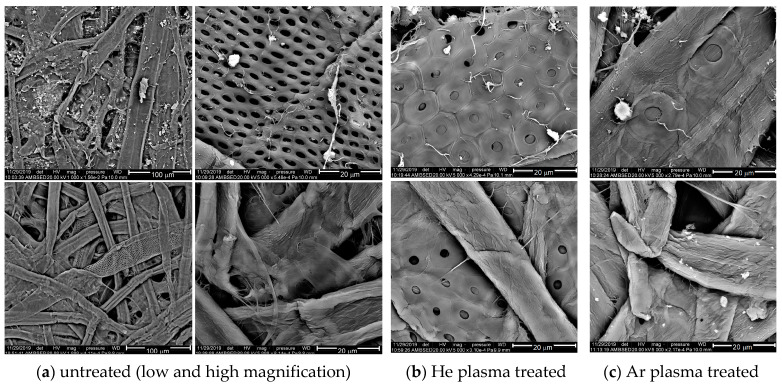
Typical SEM images of paper (**top**) and fluorinated paper (**bottom**) on untreated samples (**a**) and 120 s treated samples in He-DBD plasma (**b**) and Ar-DBD plasma (**c**).

**Figure 8:**
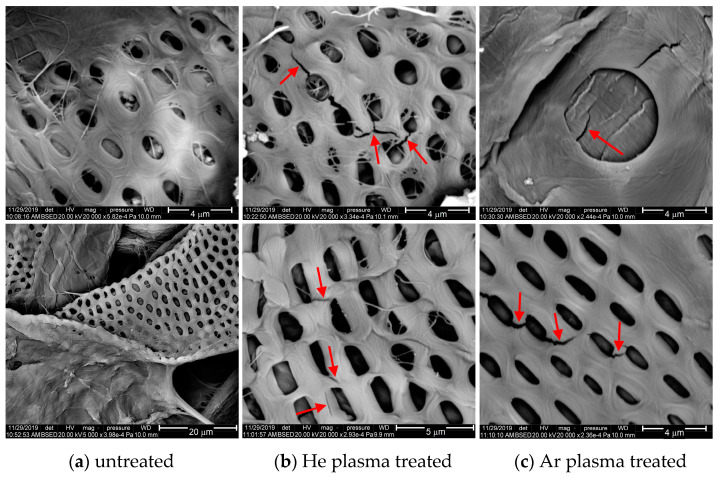
High-resolution SEM images of paper (**top**) and fluorinated paper (**bottom**) on untreated samples (**a**) and 120 s treated samples in He-DBD plasma (**b**) and Ar-DBD plasma (**c**).

The SEM images can provide valuable insights into the surface morphology of the cellulose-based surfaces (Figure 7 and Figure 8). The SEM images of the PSU films exhibit only the flat and smooth aspect of the polymer foils, with no visible features at the micrometric scale; therefore, these are not presented here.

The SEM micrographs in Figure 7a show the morphology of the untreated samples, P (top) and FP (bottom). Both cellulose-based samples have a relatively dense structure of intertwined cellulose fibers, with varying sizes. There are two main groups of cellulosic fibers. The majority group consists of fibers less than 20–25 μm thick, among which a few fibers thicker than 40 μm (but thinner than 70 μm) are embedded. In addition, the paper foils present protuberant calcium carbonate agglomerates [53], as we can see at the top of Figure 7.

Additionally, some of the thick cellulose fibers present patterns with pores, similarly distributed in two groups: oval pores with a maximum diameter of about 2 μm, positioned at a distance of 2 μm from each other, and circular pores with a diameter between 3–6 μm, situated at a distance of around 9–11 μm from each other (Figure 7 and Figure 8). 

The treated surfaces exhibit new features only for extended plasma exposure. Thus, small grooves and microcracks, which are at most a few micrometers long, appear on the surface of the cellulose fibers treated over a prolonged time (120 s) in the case of both He and Ar plasma treatments. These features are visible only on the high-resolution images and are indicated with arrows in Figure 8b,c. This aspect might result from the physical effect of the heavy particles (atoms, molecules, ions) which are present in the plasma volume and interact with the surface. Consequently, this effect produces the ablation of volatile fragments, and it eventually triggers the degradation of the polymer surface through physical sputtering and/or chemical etching in the case of longer treatment time [13,54,55].

This analysis demonstrates that the plasma used in this research has a mild effect on such sensitive material, such as paper, inducing the efficient modification of the surface properties, such as polarity, adhesion, and level of oxidation, without the alteration of the surface morphology and the physical degradation of the material, over a larger range of exposure duration. The physical effect of the plasma is visible only after a plasma exposure lasting for minutes.

Furthermore, our findings suggest that the specific behavior of liquid droplets dispensed on the treated cellulose-based surfaces, particularly the reduction in contact angle, described earlier, is not a result of the physical effect of the plasma. The accelerated diffusion of the liquid into the material may result from combined processes. These processes include the mild cleaning at the nanometric scale through removal of small volatile fragments and contaminants, and also the enhanced reactivity of the fibers’ surface by chemical functionalization, and contribute to absorbent properties. This mechanism is further substantiated by the evolution of the liquid absorption in relation to plasma exposure duration, which indicates an accelerated diffusion of the liquid drop into the polymer with longer treatment times, as explained earlier (Figure 1).

## 4. Conclusions

This study allowed us to assess the surface properties of cellulose-based polymers, such as paper (P) and fluorinated paper (FP) and synthetic polysulfone (PSU) polymer, exposed to the atmospheric-pressure DBD plasma working in He or Ar gas. The plasma treatment efficiently modifies the surface properties of the tested materials, such as wettability, adhesion, surface energy, and polarity, and also oxygen uptake. Importantly, the modified surfaces exhibit remarkable stability.

The plasma treatment induces a shift towards a hydrophilic nature in comparison to the untreated hydrophobic surfaces. Furthermore, the plasma exposure triggers absorbent properties specifically in cellulose-based materials. The initial stages of treatment (approximately 10–20 s) lead to a rapid and steep modification for all three polymers. The surface properties, including contact angle and adhesion, show a similar trend of evolution over time, tending to stabilize. The stabilization, however, occurs at different exposure times depending on the type of polymer being treated. For PSU, this level is achieved within a relatively short exposure time of about 20 s. In contrast, P and FP require a longer exposure time of approximately 120 s to reach saturation. 

After plasma treatment, the level of surface modification leads to a reversal in the ordering of the tested materials. The order of treated materials in terms of adhesion, surface energy, and polarity is P > FP > PSU, which is opposite to the ordering of the untreated materials. The materials that initially have lower adhesion, surface energy, or polarity are more responsive to enhancement in their surface properties during plasma treatment. Despite differences in their chemical structure, polarity, and surface morphology, the maximum attainable surface polarity falls within a relatively narrow interval of approximately 0.75–0.83. 

The synthetic PSU polymer exhibits a distinct behavior, reaching its maximum level of oxidation after approximately 20 s of plasma exposure. In contrast, the cellulose-based polymers, P and FP, show a continuous increase in their oxygen uptake within the range of 5 s to 120 s of exposure. Nonetheless, both P and FP reach a similar maximum oxygen content (around 40–41%), despite the differences in their oxidation mechanisms. These findings suggest that there is a limit to the extent of oxygen uptake in such cellulose-based materials. This limit is reached within the tested exposure duration range, despite these materials having a relatively high initial oxygen content.

Furthermore, the analysis of the surface morphology demonstrates that the plasma does not lead to the alteration of the surface morphology and the physical degradation of such sensitive material, such as paper, over a large range of exposure duration. 

Atmospheric-pressure plasma in an inert gas environment provides mild conditions for the efficient and stable modification of surfaces of various polymer structures. These modifications are applicable to a wide range of polymers, including those with different structural, chemical, and morphological properties. Importantly, this plasma treatment method reaches a limit of surface enhancement that is consistent across different polymer types. 

## Figures and Tables

**Table 1:** Surface tension components of the test liquids used for contact angle measurement.

Test Liquid	γ_L_ (mJ/m^2^)	γ_L_^d^ (mJ/m^2^)	γ_L_^p^ (mJ/m^2^)
Water (W)	72.8	21.8	51.0
Glycerol (G)	64.0	34.0	30.0

**Table 2:** Contact angles (CA) of water (W) and glycerol (G) measured on paper, fluorinated paper, and PSU for untreated and plasma-treated surfaces in (a) He-DBD plasma and (b) Ar-DBD plasma for various treatment times (20 s and 120 s).

Treatment Time (s)	Paper		Fluorinated Paper		PSU	
	WGA (°)	GCA (°)	WGA (°)	GCA (°)	WGA (°)	GCA (°)
0	107.4 ± 3.3	103 ± 3.0	98.8 ± 3.1	88.0 ± 3.0	83 ± 1.4	63.3 ± 1.6
(a) He-DBD plasma						
20	75.6 ± 3.3	69.5 ±6.5	52.3 ±3.2	41.0 ± 4.0	36.5 ± 2.4	32.3 ± 1.3
120	3.9 ± 1.0	9.0 ± 1.5	5.4 ± 1.0	7.5 ± 1.5	43.8 ± 1.2	38.8 ± 1.4
(b) Ar-DBD plasma						
20	57.4 ± 6.0	56.6 ± 7.2	57.6 ± 4.1	53.4 ± 7.0	38.0 ± 1.1	35.4 ± 1.7
120	3.5 ± 1.0	6.8 ± 2.0	15.5 ± 1.5	12.1 ± 1.5	39.2 ± 1.7	36.4 ± 1.0

**Table 3:** Surface polarity γ_S_^p^/γ_S_ of paper, fluorinated paper, and PSU for untreated and plasma-treated surfaces in (a) He-DBD plasma and (b) Ar-DBD plasma for various treatment times (20 s and 120 s).

Treatment Time (s)	γ_S_^p^/γ_S_		
	Paper	Fluorinated Paper	PSU
0	0.437	0.121	0.038
(a) He-DBD plasma			
20	0.529	0.504	0.748
120	0.837	0.829	0.711
(b) Ar-DBD plasma			
20	0.799	0.689	0.775
120	0.830	0.800	0.769

**Table 4:** Binding energies of the carbon functional groups in the C1s fitted spectra.

Functional Groups	Assignment		Binding Energy (eV)
	Paper/Fluorinated Paper	PSU	
carbon–hydrogen –C–C–, –C–H	C1	C1	285.0
carbon–sulphur –C–S	-	C2	285.6
carbon–oxygen –C–O–	C3	C3	286.5
carbonyl –C=O/O–C–O	C4	-	288.0
carboxyl –O–C=O	C5	C5	289.0
π−π* shake-up	-	C6	~291.8
2 fluorine–carbon –CF_2_– and 3 fluorine–carbon –CF_3_	-/C7	-	~292.3

**Table 5:** Surface total composition and atomic composition of the carbon species C1s on the paper surface (in atom %) before and after exposure in He-DBD plasma (a) and Ar-DBD plasma (b) for various treatment times.

Treatment Time (s)	Total Atomic Content (at. %)				Atomic Composition of the Carbon Groups C1s (at. %)				
	C1s	O1s	Si2p	O1s/C1s	–C–C–; –C–H	–C–O–	–C=O/ O–C–O	–O–C=O	O/C
					Binding Energy (eV)				
					285.0 ± 0.1	286.5 ± 0.1	288.0 ± 0.2	289.0 ± 0.2	
0	66.0	33.1	0.9	0.50	35.7 ± 0.2	49.3 ± 0.5	13.3 ± 0.3	1.7 ± 0.1	1.8
**(a)** **He-DBD plasma**									
5	63.8	35.3	0.9	0.55	26.4 ± 0.4	48.8 ± 1.3	20.3 ± 0.6	4.5 ± 0.1	2.8
20	61.3	37.6	1.1	0.61	24.7 ± 0.8	49.5 ± 1.1	21.5 ± 0.3	4.3 ± 0.1	3.1
120	57.6	41.3	1.1	0.72	21.4 ± 0.5	47.3 ± 1.2	23.8 ± 1.0	7.5 ± 0.2	3.7
**(b)** **Ar-DBD plasma**									
5	65.6	33.4	1.0	0.51	30.1 ± 0.4	49.5 ± 0.4	16.1 ± 0.1	4.3 ± 0.6	2.3
20	63.5	35.5	1.0	0.56	26.0 ± 0.3	48.2 ± 0.3	20.6 ± 0.2	5.2 ± 0.4	2.9
120	57.4	41.5	1.1	0.72	19.4 ± 0.4	50.3 ± 0.4	20.8 ± 0.2	9.5 ± 0.1	4.2

**Table 6:** Surface total composition and atomic composition of the carbon species C1s on the fluorinated paper surface (in atom %) before and after exposure in He-DBD plasma (a) and Ar-DBD plasma (b) for various treatment times.

Treatment Time (s)	Total Atomic Content (at. %)				Atomic Composition of the Carbon Species C1s (at. %)					
	C1s	O1s	F1s	O1s/C1s	–C–C–; –C–H	–C–O–	–C=O/ O–C–O	–O–C=O	–CF_2_–/ –CF_3_	O/C
					Binding Energy (eV)					
					285.0 ± 0.1	286.5 ± 0.1	288.0 ± 0.2	289.0 ± 0.2	~292.3	
0	53.2	27.0	19.8	0.51	18.0 ± 3.7	43.1 ± 3.4	22.8 ± 1.5	7.9 ± 1.5	8.2 ± 2.3	2.8
**(a)** **He-DBD plasma**										
5	53.2	33.4	13.4	0.63	19.3 ± 1.1	49.1 ± 0.2	21.6 ± 3.0	6.4 ± 2.0	3.6 ± 0.3	3.4
20	57.0	38.6	4.4	0.68	19.3 ± 0.7	56.0 ± 1.7	18.1 ± 2.2	5.2 ± 1.0	1.4 ± 0.3	3.8
120	57.5	41.3	1.2	0.72	19.2 ± 0.1	59.3 ± 0.1	17.4 ± 2.1	4.1 ± 2.0	0	4.2
**(b)** **Ar-DBD plasma**										
5	54.7	30.0	15.3	0.55	17.2 ± 2.1	54.3 ± 3.6	16.5 ± 3.1	3.6 ± 0.9	8.4 ± 0.1	2.9
20	52.9	33.2	13.9	0.63	18.9 ± 0.3	44.4 ± 1.8	26.0 ± 0.9	7.0 ± 0.3	3.7 ± 0.4	3.4
120	56.2	40.0	3.8	0.73	17.2 ± 0.8	56.3 ± 0.6	21.0 ± 0.7	5.5 ± 0.5	0	4.8

**Table 7:** Surface total composition and atomic composition of the carbon species C1s on the PSU surface (in atom %) before and after exposure in He-DBD plasma (a) and Ar-DBD plasma (b) for various treatment times.

Treatment Time (s)	Total Atomic Content (at. %)				Atomic Composition of the Carbon Species C1s (at. %)					
	C1s	O1s	S2p	O1s/C1s	–C–C–; –C–H	–C–S	–C–O–	–O–C=O	π−π* Shake-Up	O/C
					Binding Energy (eV)					
					285.0 ± 0.1	285.6 ± 0.1	286.5 ± 0.1	289.0 ± 0.2	~291.8	
0	80.9	16.4	2.7	0.20	75.2	7.1	14.3	-	3.4	0.17
**(a)** **He-DBD plasma**										
5	74.1	23.5	2.4	0.32	65.4	6.9	21.7	3.6	2.4	0.34
20	71.0	26.8	2.2	0.38	58.6	9.5	21.9	7.1	2.9	0.41
60	72.2	24.7	3.1	0.34	61.5	11.6	18.0	4.9	4.0	0.30
120	74.4	22.9	2.7	0.31	63.1	12.1	17.7	3.6	3.5	0.27
**(b)** **Ar-DBD plasma**										
5	73.6	23.8	2.6	0.32	59.6	10.9	20.3	5.8	3.4	0.35
20	71.5	25.9	2.6	0.36	59.0	10.3	21.4	6.5	2.8	0.39
60	73.0	24.0	3.0	0.33	61.5	11.0	20.0	3.2	4.3	0.30
120	75.2	22.1	2.7	0.29	62.1	12.8	18.5	3.2	3.4	0.28

## Data Availability

The data presented in this study are available on request from the corresponding author.
